# “It was with my consent since he was providing me with money”: a mixed methods study of adolescent perspectives on peacekeeper-perpetrated sexual exploitation and abuse in the Democratic Republic of Congo

**DOI:** 10.1186/s13031-021-00414-0

**Published:** 2021-11-06

**Authors:** Georgia Fraulin, Sabine Lee, Sandrine Lusamba, Susan A. Bartels

**Affiliations:** 1 Faculty of Health Sciences/Biomedical and Molecular Sciences, Queen’s University, Kingston, Canada; 2 Department of History, University of Birmingham, Birmingham, UK; 3 Solidarité Féminine Pour La Paix et le Développement Intégral, Beni, Democratic Republic of Congo; 4 Departments of Emergency Medicine and Public Health Sciences, Queen’s University, Kingston, Canada

**Keywords:** Democratic Republic of Congo, MONUSCO, Peacekeeping, Sexual abuse and exploitation, Adolescents, Women and girls, United Nations, Transactional sex, Protection

## Abstract

**Background:**

The United Nations Organization Stabilization Mission in the Democratic Republic of Congo (MONUSCO) has been marred by widespread allegations of sexual exploitation and abuse (SEA) of women and girls by UN peacekeepers. There is minimal research conducted on the perceptions of communities most affected by the SEA themselves, and even less about those below the age of 18.

**Methods:**

Using mixed-methods data, we examined the perceptions of adolescents aged 13–17 on how the lives of women and girls have been affected by the presence of UN peacekeepers within the DRC. SenseMaker, a mixed-methods narrative capture tool, was used to survey participants around six United Nations bases across eastern DRC. Each participant shared a story about the experiences of Congolese women and girls in relation to MONUSCO personnel and interpreted their own stories by answering a series of questions. Patterns of adolescent perspectives (aged 13–17) were analyzed in comparison to all other age groups and emerging qualitative themes were mapped onto quantitative variables.

**Results:**

Quantitative data showed that adolescents were more likely, in comparison to all other age groups, to perceive interactions between peacekeepers and women/girls as being initiated by the woman/girl, that the MONUSCO personnel was perceived to be able to offer protection, and that the interactions between local women/girls and peacekeepers were sexual in nature. Three qualitative themes emerged: poverty bringing peacekeepers and women/girls together, material/financial gain through transactional sex and sex work, and support-seeking actions of affected women/girls.

**Conclusions:**

Our mixed methods data illustrate the problematic finding that adolescents facing poverty may perceive SEA as protective through the monetary and material support gained. These findings contribute to the growing body of literature on peacekeeping economies and have implications for the prevention of, and response to, peacekeeper-perpetrated SEA.

## Background

The Democratic Republic of Congo (DRC) is a sub-Saharan African country with high poverty rates and a low human development index. The post-independence Zairian period from 1965–1997 was succeeded by the Congolese civil wars between 1996–97 and 1998–2003 which intertwined international and regional conflicts [1, 2]. The origins of these conflicts lie in part with the massive refugee crisis that resulted from the 1994 Rwandan genocide [3]. The country has faced decades of ongoing conflict which has killed more than 5 million people [3]. Furthermore, conflict has continued surrounding political leaders, various militias, and control of mineral resources [4, 5].

Widespread sexual and gender-based violence (SGBV) has been pervasive during the ongoing conflicts within the DRC. Rape during conflict has been called a “weapon of war” [6] and can be associated with extreme violence [7]. However, non-conflict related SGBV is also highly prevalent in DRC. In 2011, it was estimated that 1150 women were raped per day in the DRC [8]. The vulnerability of women to violence is increased by poverty, and in turn violence reduces women’s capacity to engage in income-generating activities to reduce poverty [9].

### Peacekeeping in the DRC

The international community, and specifically, the United Nations (UN), have been involved in peacekeeping operations (PKO) in the DRC since 1998 [1]. The initial peacekeeping mission from 1999-June 2010 was called the Mission de l’Organisation des Nations Unies en République Démocratique du Congo (MONUC) [10]. MONUC was one of the largest missions ever deployed with 20,586 uniformed personnel at its peak [10]. MONUC transformed into the United Nations Organization Stabilization Mission to the Mission de l’Organisation des Nations Unies pour la Stabilisation en République Démocratique du Congo (MONUSCO) in May 2010 [11]. Currently consisting of over 16,500 personnel [12], MONUSCO’s mandate is to protect civilians and to contribute to the stabilization and consolidation of peace [13].

### Sexual exploitation and abuse in United Nations peacekeeping missions

First reported in the early 1990s, sexual exploitation and abuse (SEA) of civilians by UN peacekeepers is now recognized as an endemic issue within PKOs. Sexual exploitation is defined by the UN Secretariat (2003) as “any actual or attempted abuse of a position of vulnerability, differential power, or trust, for sexual purposes, including, but not limited to, profiting monetarily, socially or politically from the sexual exploitation of another” [14]. Sexual abuse is “the actual or threatened physical intrusion of a sexual nature, whether by force or under unequal or coercive conditions” [14]. Peacekeeper-perpetrated exploitation and abuse includes sexual assault, trafficking, forced prostitution, child pornography, abduction, transactional sex, and networked SEA [15–32]. Transactional sex, or “survival sex” includes the exchange of sex for money, food, or jobs and includes some degree of agency [17]. Networked SEA involves the trafficking of women or children, whether through forced prostitution, or the expansion of the sex industry, including brothels [17]. The consequences of SEA-related misconduct within PKOs goes beyond the mistreatment of women and children and also negatively impacts the UN’s and missions’ credibility, with the UN compromising its moral standing on human rights issues and international rule of law [15].

Both MONUC and MONUSCO have been marred by widespread allegations of SEA against local women and girls by UN peacekeepers [15, 22–26]. Allegations of peacekeeper-associated SEA by MONUC peacekeepers first arose in 2004 via a number of news reports alleging 150 cases of sexual assault, including 68 cases of rape, prostitution and paedophilia, as well as cases of torture, child pornography and the fathering of peacekeeper-fathered children [22, 23, 25, 26]. The majority of SEA allegations within the Congolese PKOs involved sex with individuals under the age of 18 [15], with transactional sex being particularly prevalent [33]. Collectively, MONUC faced 181 SEA allegations between 2007 and 2010, while between 2010 and 2021 MONUSCO has incurred 224, which is significantly higher than other PKOs [34].

Peacekeeper-perpetrated SEA has sometimes been explained by militarized masculinities, in which peacekeepers see themselves as protectors of fragile populations, in particular women and children, which influences their conceptions of masculinity [35–37]. The low-combat nature of most peacekeeping missions may challenge the masculinity of peacekeepers and minimize their identity as soldiers, which may contribute to the use of sexual abuse to “express their masculinity” [38]. Similarly, Kovatch [37] states that peacekeepers suffer crises of masculinity on missions due to the paradox of being “warriors sent to ‘wage peace’”. However, notably, Higate [28] rejects the overemphasis of militarized masculinities in favour of investigating how socioeconomic structure, privilege and impunity influence exploitative and oppressive social masculinities of certain male peacekeepers. Additionally, poverty, the volatility that necessitates a peacekeeping operation, and country-level gender equities intersect with the SEA of women and girls by UN peacekeepers. Kent (2005) concluded that widespread conflict and displacement weaken social, economic, and political structures, resulting in the lack of capacity to address and protect civilians against sexual abuse [15].

### Accountability and responses from the UN

Although allegations of peacekeeper-perpetrated SEA began in the early 1990s [31, 39, 40], it was 2002 before the UN began to address the issue. Following the Secretary General’s Bulletin on “Special Measures for Protection from Sexual Exploitation and Sexual Abuse” established in 2003 by the Inter-Agency Standing Committee (IASC) Task Force on Protection from Sexual Exploitation and Abuse in Humanitarian Crises [14], in 2005, the “Comprehensive Strategy to Eliminate Future Exploitation and Abuse in United Nations Peacekeeping Operations” [18], or the “Zeid Report,” initiated what has since become known as the UN’s zero-tolerance policy. The Zeid Report prompted a number of documents and initiatives to prevent SEA, including consequences for peacekeeping personnel who perpetrate SEA [41]. Two notable resolutions have been adopted by the UN General Assembly: Resolution 62/63, which outlined criminal responsibility [42], and Resolution 62/214, which outlined the “Comprehensive Strategy on Assistance and Support to Victims of Sexual Exploitation and Abuse by United Nations Staff and Related Personnel” [43].

These resolutions and policies have been regarded as inadequate both with respect to responding to victims as well as punishing perpetrators [41, 44]. The most prominent issue with holding individual peacekeepers accountable is their functional immunity, which prevents them from being directly prosecuted for crimes committed while engaged in official peacekeeping duties. While military personnel are bound by Memorandums of Understanding and the Status of Forces Agreements, and these agreements technically expect troop contributing countries to hold their troops accountable, there is no legal obligation to do so [24, 45]. Martuscelli & Rinaldi (2017) state that this creates an “impunity environment” as personnel are aware that repatriation is the worst that can happen [35].

## Aims and rationale

With the exception of [46], empirical evidence on adolescent and child perspectives about peacekeeper-perpetrated SEA in the DRC is lacking [46] suggests that children and adolescents, mostly young girls, are particularly affected by peacekeeper-perpetrated SEA [46] A limited number of studies have been published about the local perceptions of women and girls in Haiti regarding the United Nations Stabilization Mission in Haiti (MINUSTAH) [30, 47–49]. While not limited to peacekeeper-perpetrated SEA, adverse psychological effects and health outcomes such as post-traumatic stress disorder, depression, long-term fear, anxiety, insomnia, low self-esteem, and social withdrawal are experienced particularly severely in children [50]. Furthermore, underage girls cannot consent to sexual interactions with adult men such as male peacekeepers [17]. Therefore, this study aims to understand the unique perceptions and vulnerabilities of adolescents by presenting a mixed quantitative–qualitative analysis of local adolescent perceptions of interactions between MONUSCO personnel and Congolese women/girls.

## Methods

### Study design and participants

This is a mixed-methods analysis of cross-sectional study data collected in 2018 using Cognitive Edge’s narrative capture tool, SenseMaker. Six UN bases across eastern DRC were purposively chosen for data collection based on size, location, staffing by national militaries, and dates of operation. A convenience sample of prospective participants was then approached in naturalistic settings such as shops, markets, parks, and transportation depots in the six locations: Bukavu, Kalemie, Goma, Kisangani, Beni and Bunia. Individuals 13 years of age or older were eligible for participation.

**Fig. 1:**
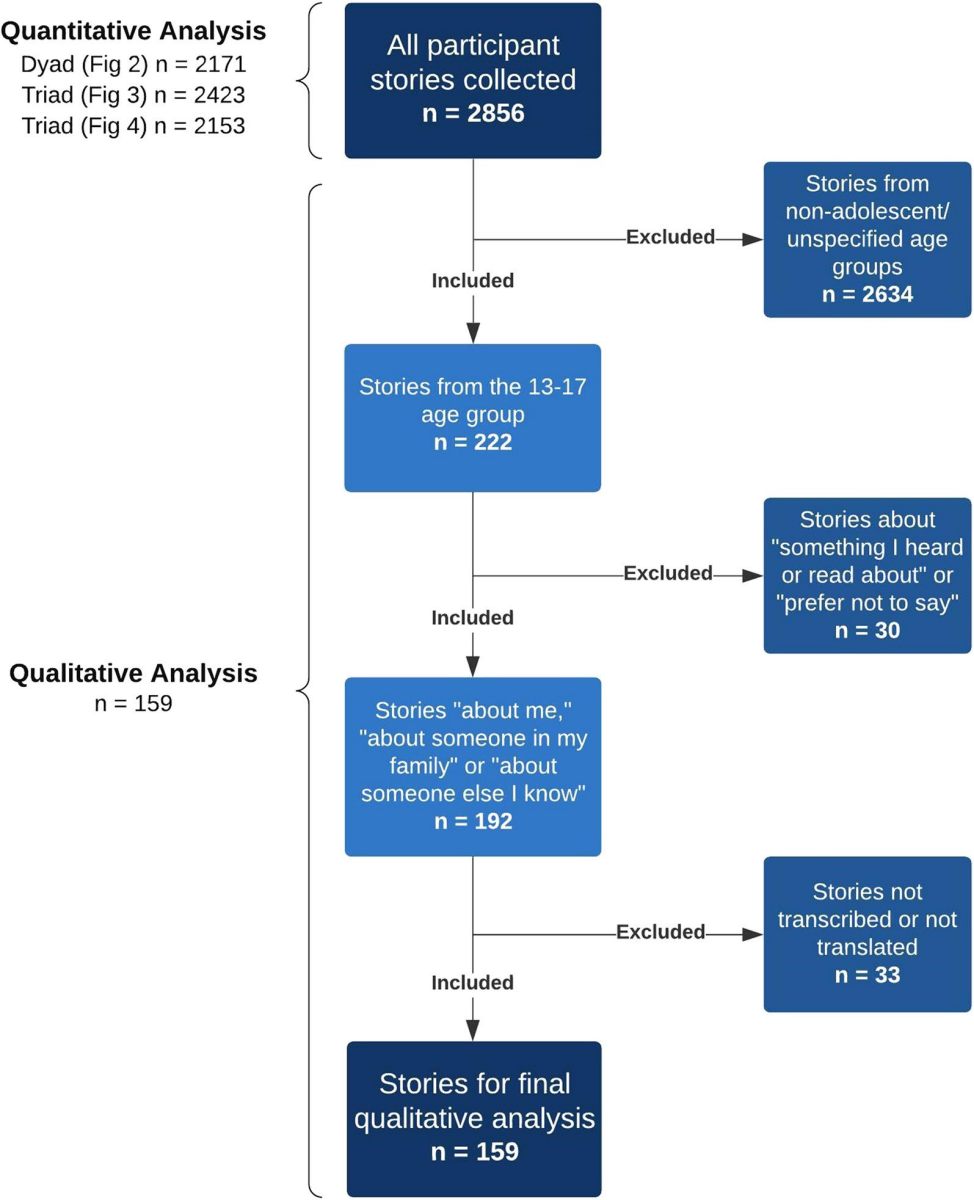
Mixed-methods analysis process. Note: All quantitative dyad and triad questions were optional, resulting in the number of respondents varying by each quantitative question

As shown in Fig. 1, our quantitative analysis used data from all age groups and compared adolescents aged 13–17 (n = 222) with all other age categories (n = 2634). Transcribed narratives from adolescents aged 13–17 who shared a first-person narrative, a narrative about someone in their family or someone they knew (n = 159) were then thematically analyzed to help explain and contextualize the quantitative findings.

### Data collection instrument

Cognitive Edge’s SenseMaker is a mixed-methods narrative capture tool. It extracts meaning from “micronarratives” shared by study participants regarding a topic of interest, in this case, the experiences of local women/girls interacting with UN peacekeepers. See Appendix 1 for full narrative prompts and survey questions. The survey did not ask directly about SEA or outcomes of sexual interactions between peacekeepers in women/girls. This was intentional to prevent reporting biases that can occur when the research is limited to a narrow research hypothesis [51]*,* and to allow stories to emerge from the lived experiences of participants.

The survey was created by research team members with expertise encompassing SGBV, humanitarian crises, and peacekeeping alongside a SenseMaker consultant. It was first written in English, then translated to Swahili and Lingala and back translated to ensure accuracy. Discrepancies in translation were resolved by consensus. The SenseMaker survey was pilot tested with 24 participants in DRC to check for clarity, translation accuracy, and ease of response.

After the narratives were audio recorded, participants interpreted their shared experiences by responding to a series of predefined questions in the form of dyads (choosing between two possible responses on a sliding scale) and triads (choosing between three possible responses within a triangle) using handheld tablets. The full survey is provided in Appendix 1. Multiple-choice questions collected demographic information and data to contextualize various aspects of the narrative.

### Study implementation

Twelve trained Congolese research assistants were purposively selected from our local partners, Solidarité Féminine Pour la Paix et le Développement Intégral (SOFEPADI) and the Marakuja Kivu Research Group. SOFEPADI is a non-governmental organization (NGO) devoted to promoting the rights of women and Marakuja is a local research organization. Before data collection, research assistants were trained in research ethics, confidentiality, informed consent, data entry, SenseMaker methodology, sampling, reporting adverse events, the referral process for counseling or services, and security protocols. Single, face-to-face interviews were conducted by the research assistant team. In response to a prompting question, participants shared a micronarrative which was audio recorded in Swahili or Lingala and later transcribed plus translated to English by a professional Congolese translator. SOFEPADI team members guided cultural and ethical considerations for the study’s implementation.

### Ethical approval

The Queen’s University Health Sciences and Affiliated Teaching Hospitals Research Ethics Board (protocol #6020398) and the Congolese National Committee of Health Ethics (CNES 001/DP-SK/119PM/2018) approved this study. All interviews were conducted confidentially, and all data were de-identified from the point of collection. Consent and study information were reviewed with each individual participant by trained research assistants in Swahili or Lingala. Participants indicated their consent by ticking a consent box on the tablet.

The youngest group of participants interviewed (the focus of this study) were aged 13–17. The risk of participating in this study was regarded as minimal given that it did not mention or explicitly ask about sexual interactions. Furthermore, we considered the adolescents to be mature minors and asking parents for consent may have led to intra-family conflict and introduced bias [52]. Thus, parental consent was waived by the ethics boards. No monetary or other compensation was provided to participants.

### Analysis

#### Quantitative analysis

Triad and dyad data were exported to Tableau (V.2020.4) and patterns of perspectives were identified from the collective plots [53]. Questions that appeared to have visual differences between age sub-groups were selected for statistical analysis to understand how adolescents responded in comparison to older age groups.

Dyad data is presented in the form of histograms divided into eleven bars of equal width. The three bars at the most extreme ends of the histogram were combined to summarize response patterns. SPSS (IBM SPSS Statistics V.24.0.0.0) was used to analyze dyad responses via the Kruskal–Wallis H test with a chi-squared test statistic to determine if the area under those extreme three bars differed statistically between age groups [54, 55]. Dunn’s Test was used in post-hoc analysis to analyze which sub-groups differed from each other. Differences were declared statistically significant with *p*-values < 0.05.

For triad data, R scripts (R V.3.4.0) was used to generate geometric means and 95% confidence intervals for each subgroup. Confidence intervals are represented as confidence ellipses around the geometric means [56]. The geometric mean for a particular subgroup was determined to be statistically different if its 95% confidence ellipse did not overlap or touch the confidence ellipses of other subgroups.

#### Qualitative analysis

To understand why adolescents aged 13–17 were statistically more likely to respond differently on several of the triad/dyad interpretation questions compared to other participant age groups, we then conducted a qualitative analysis of the adolescents’ micronarratives (n = 159).

Thematic analysis was conducted according to Braun and Clarke [57]. At the first level of analysis, a codebook was developed by GF and SB according to DeCuir-Gunby, Marshall & McCulloch [58]. The codebook contained both theory-driven and data-driven codes derived from the pertinent literature and the transcripts, respectively. Using the codebook and Dedoose (V 8.3.43), GF led narrative coding and SB co-coded ~ 20% of narratives. In the second level of analysis, codes were organized into pertinent themes, or categories, that reflected the meanings and experiences shared in the narratives. The themes were not mutually exclusive, and stories were placed into more than one theme where appropriate. Salient quotes from each theme were selected through consensus to illustrate the diverse perspectives of adolescents about MONUSCO peacekeepers.

The researchers engaged in critical dialogue throughout the analysis. Memos and notes were used as an audit trail for all levels of coding and the team engaged in constant comparison to assess each code and narrative in relation to all other data, in addition to evaluating each narrative’s individual information.

## Results

Knowing from previous literature that adolescents were particularly affected by peacekeeper-perpetrated SEA [15], we examined the triad and dyad data by age group for the total sample of 2856. Adolescent perspectives were statistically different from all other age groups on 2 triads and 1 dyad.

### Dyad

**Fig. 2:**
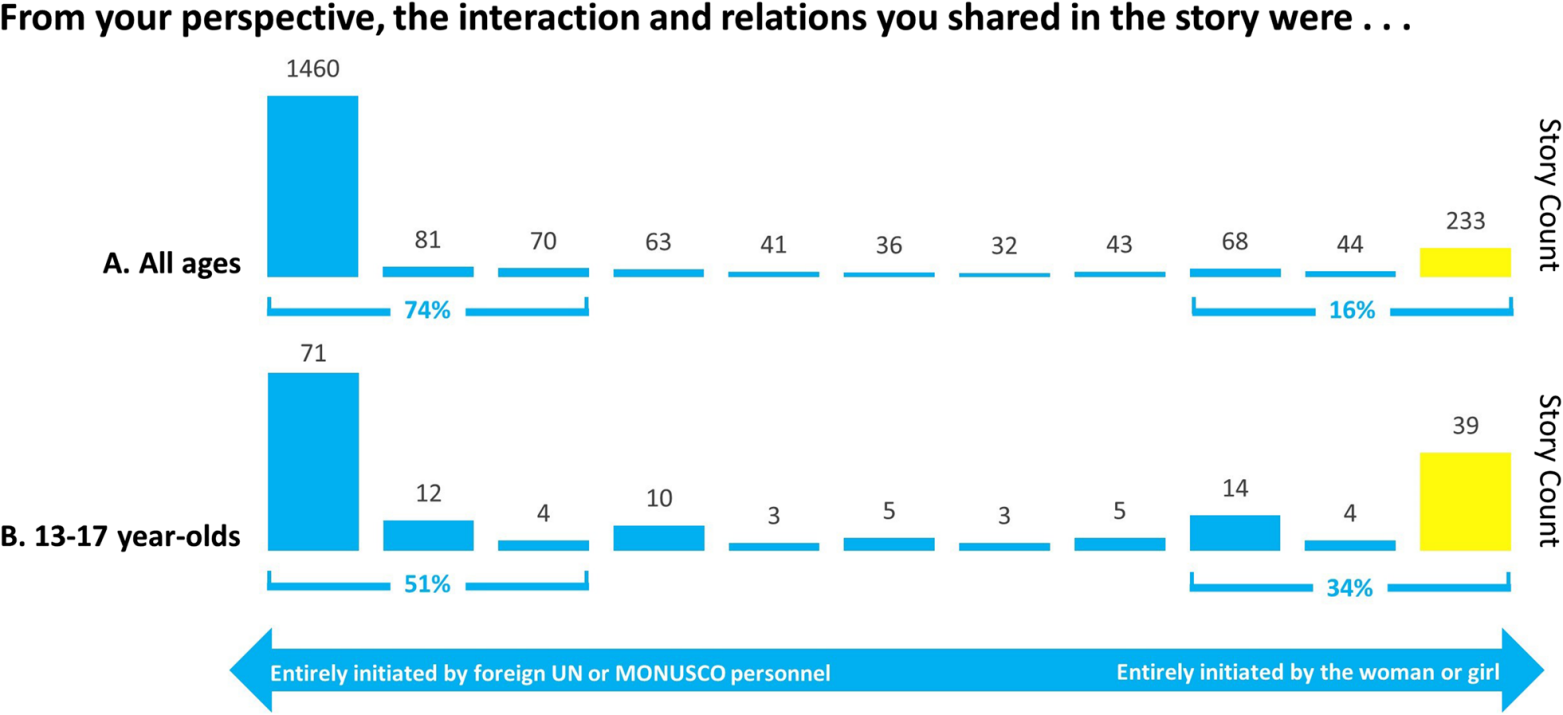
Dyad regarding initiation of the shared interaction. (**A**) All ages except 13–17 (n = 2171). (**B**) 13–17 year-olds (n = 170) (*p* < 0.001)

Dyads allowed participants to interpret the experiences of the women/girls in their stories by sharing their perspectives between two extremes. The dyad in Fig. 2 asked who had initiated the interaction in the narrative (Fig. 2). An independent-samples Kruskal–Wallis H test showed a statistically significant difference in patterns of response across age sub-groups, χ^2^ = 93.615, *p* < 0.001. In post hoc analysis, responses for the 13–17 age group differed from all other age groups, with the 13–17 age group being more likely to report that the interaction was entirely initiated by the woman or girl in the story (all *p*-values < 0.001). While overall, women were more likely to indicate that the interaction was entirely initiated by the woman/girl in the story (*p* < 0.001), there were no statistically significant differences in response patterns between males and females within the adolescent group aged 13–17 (*p* = 0.179).

### Triads

**Fig. 3:**
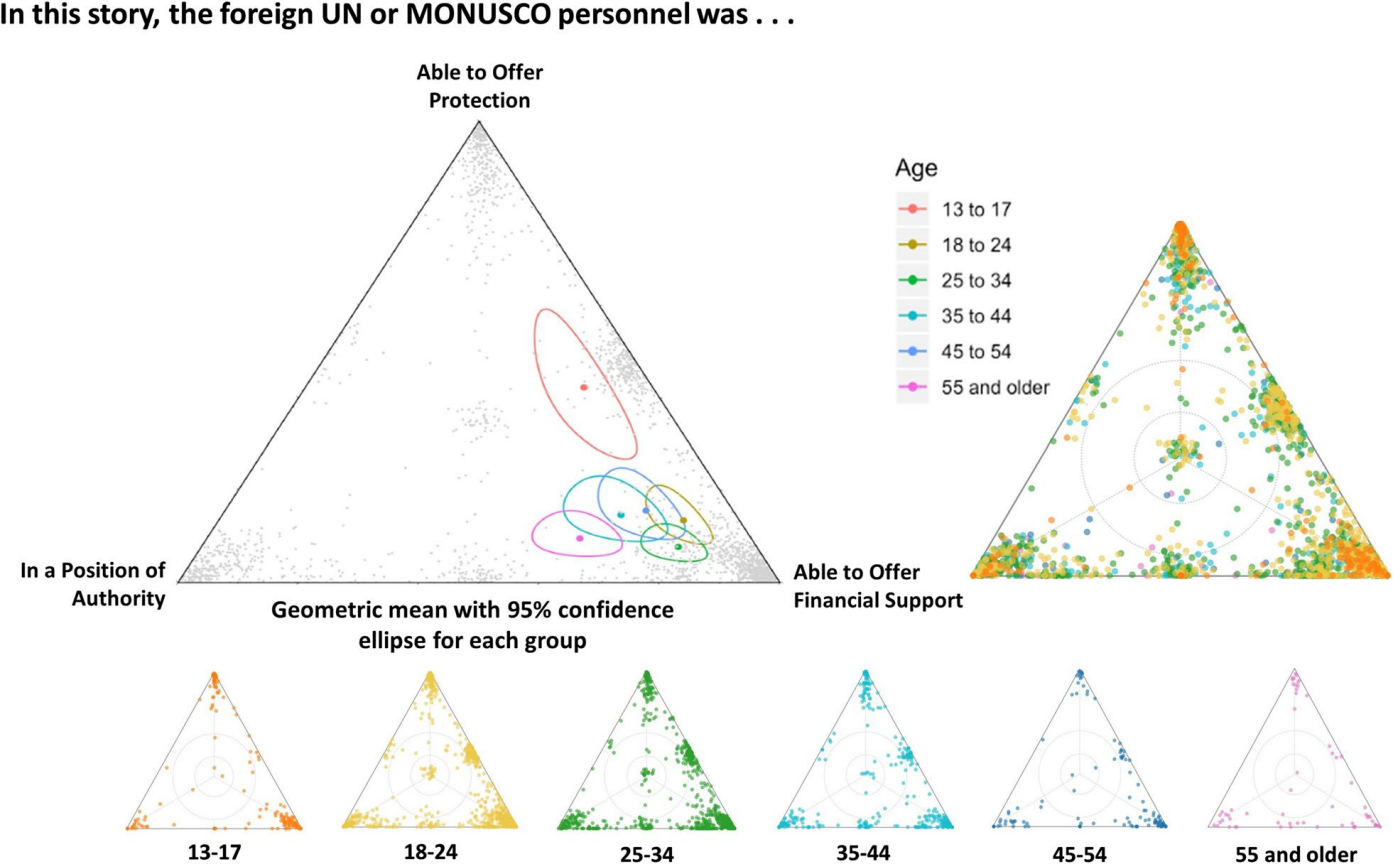
Triad regarding the positionality of the UN/MONUSCO personnel in the shared story (n = 2423). The 13–17 age group confidence ellipse does not overlap with other 95% confidence ellipses, indicating the geometric mean for the 13–17 group is statistically different. When disaggregated by gender, male adolescents are shown to be responsible for this statistically significant result

Triads allowed participants to interpret the shared experiences between three predetermined labels on a triangle. Each dot within a triangle represents a narrative, colour-coded according to age subgroup. The geometric mean for each age subgroup is represented as a point with a 95% confidence ellipse surrounding it. The triad in Fig. 3 asked about the positionality of the UN or MONUSCO personnel in the shared narrative. The geometric mean for the 13–17 age group differed significantly from all other groups as illustrated by its non-overlapping confidence ellipse. The 13–17-year-old participants were more likely than all other age groups to perceive that the MONUSCO personnel was able to offer protection. From our team’s perspective, “protection” was intended to refer to improved safety or security. However, as per SenseMaker’s strategic design, protection was not defined in the survey. Instead, participants were allowed to interpret protection according to their own opinions and viewpoints, which are then revealed through analysis of the accompanying micro-narratives. When the data were further disaggregated into age and gender, it was clear that male adolescents were primarily driving the overall result of participants aged 13–17 being more likely to indicate that the UN personnel was offering protection.

**Fig. 4:**
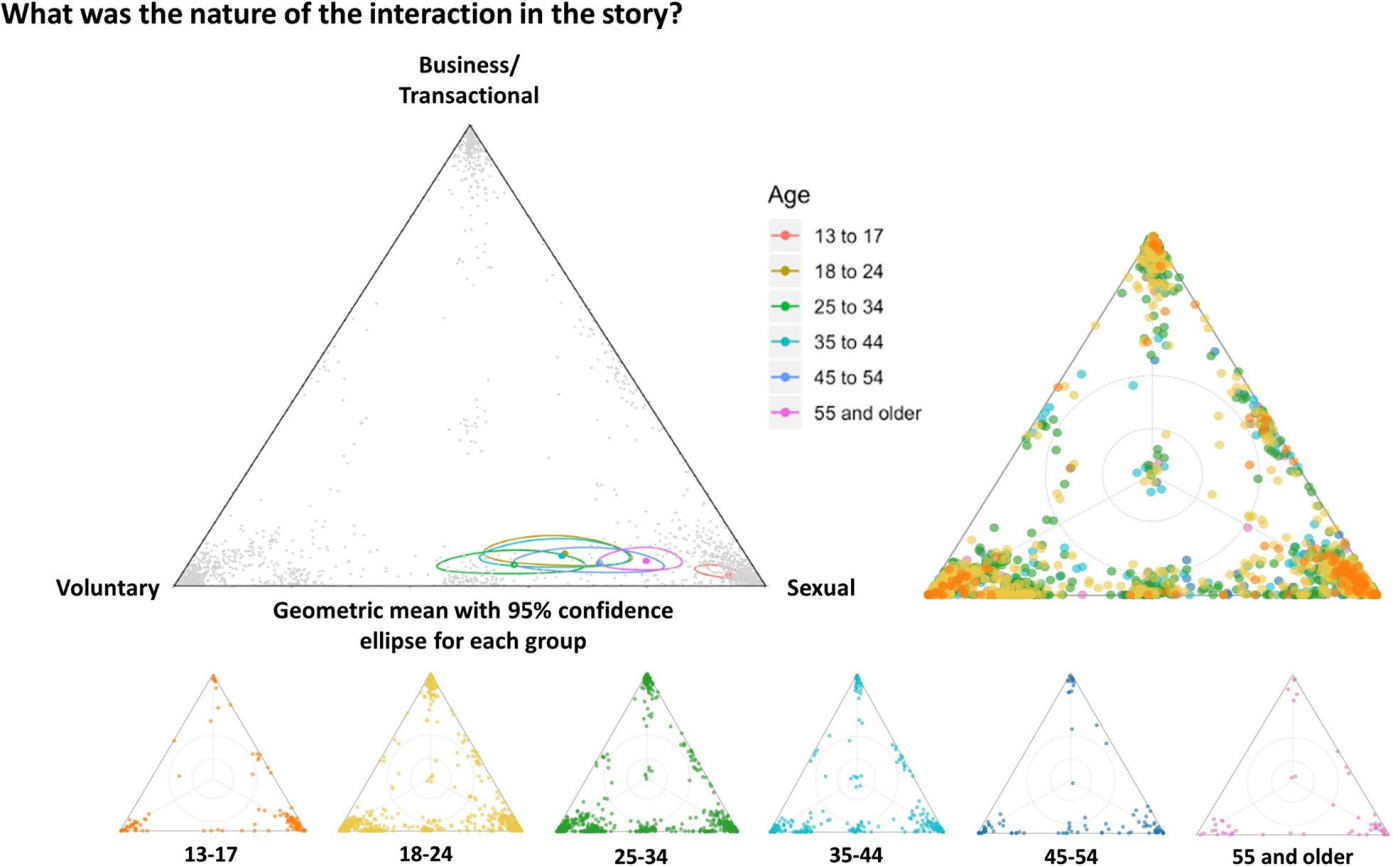
Triad regarding the nature of the interaction with the UN/MONUSCO personnel (n = 2153). The 13–17 age group’s confidence ellipse does not overlap with other 95% confidence ellipses, indicating that adolescent participants responded in a statistically significant different way. The geometric mean for males aged 13 – 17 is represented by the red dot in the vertex labelled ‘sexual’. Given the smaller n for male adolescents (n = 80), a 95% confident ellipse could not be generated for this geomean

The triad in Fig. 4 asked about the nature of the interaction between the woman/girl and the UN peacekeeper in the shared narrative. The geometric mean for the 13–17 age group differed significantly from all other groups as illustrated by its non-overlapping confidence ellipse. Participants aged 13–17 being more likely to interpret the nature of the interaction in the story as sexual, rather than business/transactional or voluntary. When these data were disaggregated by gender, it revealed that male adolescents were again largely responsible for this statistically significant finding.

### Qualitative themes

To contextualize why adolescents were statistically more likely to report that interactions with peacekeepers were sexual in nature, to examine how peacekeepers were perceived to offer protection, and to understand why adolescents were more likely to report that interactions with peacekeepers were initiated by the woman/girl, we qualitatively analyzed adolescents’ narratives (n = 159). Demographics for the analyzed adolescent micronarratives is provided in Table 1.

**Table 1:** Participant demographics of qualitative micronarratives analyzed

Demographic	Value	n	%
Age	13–17	**159**	**100**
Who story is about	About me	26	16.35
	About someone in my family	26	16.35
	Someone else I know	107	67.30
Sex	Male	59	37.11
	Female	100	62.89
Income group*	Average	83	52.20
	Poor	61	38.36
	Well-off	15	9.44
Education	Some secondary school	53	33.33
	Some primary school	62	38.99
	None	19	11.95
	Completed primary school	14	8.81
	Some university	4	2.52
	Completed secondary school	6	3.77
	Completed technical training	1	0.63
Marital status	Single never married	152	95.60
	Divorced	0	0.00
	Living with partner	1	0.63
	Married	6	3.70
	Prefer not to say	0	0.00
	Separated	0	0.00
Area collected	Bukavu	46	28.93
	Kalemie	13	8.18
	Goma	93	58.49
	Kisangani	3	1.89
	Beni	2	1.26
	Bunia	2	1.26

*We assessed household income through a proxy measure which asked if the family owned any the following items: (1) mobile phone, (2) radio, (3) generator inverter or sun panel, (4) any type of motorized vehicle, or (5) refrigerator or freezer. Participants could choose as many items as was applicable or they could choose ‘none of the above.’ Income was then categorized as follows: access to none or 1 of the 5 items was rated as ‘poor,’ access to 2 or 3 of the items was rated as ‘average’ and access to 4 or 5 of the items was rated as ‘well-off.’

Three broad themes emerged to better understand the quantitative results: poverty bringing the peacekeeper and woman/girl together, material/financial gain through transactional sex and sex work, and support-seeking actions of affected woman/girls.

1. *Poverty*

Poverty was often a key contributor to the initiation of the interaction between the peacekeeper and local woman/girl. Within the analyzed micronarratives, the three most common financial or material needs being fulfilled through sexual interactions with peacekeepers were: (1) Need for/lack of education, (2) Food insecurity/need for material goods, and (3) Taking care of family.

#### Need for/lack of education

A subset of narratives described the need for, or lack of, girls’ education as a motivating factor for interacting with MONUSCO personnel. For example, the following participant interpreted the interaction as being initiated by the woman/girl, the type of interaction as sexual, and the peacekeeper as being able to offer protection. He discusses a young woman being “friends” with a MONUSCO agent who helped pay her school fees:

*The young lady was a friend of a Uruguayan of MONUSCO. She was loved by him and presently she has no child, but they continue to be friends. They are only helping her to pay school fees. (Male participant ID485)*

Additionally, this participant who interpreted the peacekeeper as being able to offer protection, shared a narrative about not being able to pay her school fees, and as a result spent time at a MONUSCO base which resulted in a pregnancy:

*I was a pupil; I did not keep up with my studies because of not being able to pay. Due to that, I was spending my day at the camp of MONUSCO. As I used to be there at the camp of Tanzanians. One MONUSCO agent fell in love with me… Finally, I became pregnant of him, and the man went away. (Female participant ID 96)*

#### Food insecurity/need for material goods

Another subset of narratives focused on how poverty manifested as food insecurity. Participants spoke about how a lack of food led adolescents to spend time at MONUSCO camps, for example by stating “*There are some impossible children who abandon schools because of going to collect waste foods at MONUSCO*” (Female participant ID 863).

Furthermore, food was used to coerce women/girls into having sex with UN peacekeepers, as mentioned in narratives such as the following:

*On my way from the church for prayers, I saw a Tanzanian… He called and said that he loved me. He courted me and distracted me with pieces of bread and biscuit and slept with me. (Female participant ID 320)*

A few narratives also described how sex workers were given food, including “young ladies” who were “prostituted” to receive food for sexual intercourse. The peacekeeper in the following narrative was perceived as being able to offer protection:

*They prostituted local young ladies. When they met them, they got packs of bread… Even young ladies, who were virgins, entered the brothels and got pieces of bread for the sexual intercourse. (Female participant ID 1923)*

#### Taking care of family

Poverty also manifested as the need for women/girls to help take care of their families in a way more concretely stated than the need to fulfil basic needs such as food as in the previous subtheme. In some cases, furniture, appliances, and so on were provided by the peacekeeper to the woman/girl’s family. For example, in the following narrative, interpreted as being mostly sexual in nature, a participant spoke about how a peacekeeper was helpful to the family because of his ability to provide material goods.

*A story that I have is about a girl from my family who fell in love with a MONUSCO agent. That man was good to us, he was considered as one of our family since he was buying us some good things that no one could imagine. We were actually okay with him. (Female participant ID 1132)*

2. *Material/financial gain through transactional sex and sex work*

In the context of extreme and pervasive poverty, many adolescents referred to compensation (monetary or otherwise) in their descriptions of the interactions between the women/girls and MONUSCO personnel. Our analysis identified three sub-themes: (1) Transactional sex, (2) Sex work, and (3) Intermediary who facilitated the interaction. Each theme with illustrative quotes is presented below.

#### Transactional sex

Transactional sex was coded according to Stoebenau et al. (2016)’s three paradigms: sex for basic needs, sex and material expressions of love, and sex for improved social status [59]. Of these, sex for basic needs was the most prominent in our analysis. Narratives within this sub-theme shed some light on how transactions were interpreted as being initiated by women/girls. For example, the girls in the following narrative were perceived to have initiated the interaction which involved showing their breasts in exchange for gifts, food and money:

*The guys of MONUSCO give money to girls who accept to show them their breasts…When the chief of is absent they do stupid things like asking girls to show their breasts in exchange with gifts, food, money. (Female participant ID 236)*

Although the notion of consent was raised by certain adolescent participants, it is important to note that even if the adolescent perceived the interaction to be consensual, consent is rendered immaterial in cases of sexual intercourse with underage minors since it constitutes statutory rape. For example, consent was mentioned in this first-person narrative from a girl who reported that the UN personnel was able to offer financial support:

I was going out with a MONUSCO agent… He was not forcing me to have sex with him, but it was with my consent since he was providing me with money. (Female participant ID 1512)

Stoebenau’s transactional sex paradigms of sex for basic needs as well as sex and material expressions of love [59] emerged from narratives where the relationship was suggested to be loving, such as the following narrative in which the participant interpreted the interaction as sexual:

*That MONUSCO agent was a teacher, we were loving each other, he used to come home when we were loving each other, while staying, he could come and bring me bread or money so that I can buy what I need only. (Female participant ID 47)*

Narratives also shed light on how sexual interactions that involved financial or other compensation from peacekeepers were perceived as protective, with peacekeepers doing good things. For example, the UN peacekeeper in the following interaction, which was perceived to have been initiated by the woman/girl, was viewed as offering protection:

*…In most cases, MONUSCO is doing something great. As far as sexual abuse is concerned, I know that girls always go to the airport to meet up with MONUSCO agents. So, they enter there with a view to having intimate relations with them. (Male participant ID 2823)*

Additionally, there was also a belief among some participants that relations with foreign peacekeepers would “improve” the lives of women/girls. A male participant who perceived that the peacekeeper in his shared narrative was able to offer protection stated, “Girls think they *get salvation* when they enter MONUSCO compound” (ID 2822). A similar sentiment was articulated in the following narrative that referenced “going out” with foreign peacekeepers to improve one’s life. The peacekeeper in this interaction was viewed by the participant as being able to offer protection:

*And as you can see, all those women who are used to going out with MONUSCO guys haven’t had their lives improved at all. You will always see them idling into pubs and clubs like DE LA PAIX, MA COLLINE, receiving MONUSCO guys and other men there, but their lives haven’t changed. (Male participant ID 2050)*

#### Sex work

Commercial sex work was mentioned often within the narratives, often articulated as “prostitutes” who were perceived to be protected through the financial resources provided by MONUSCO personnel. For example, the peacekeeper in the following narrative was interpreted by the participant as being able to offer protection and financial support:

*Most of those women who always meet with MONUSCO guys are real prostitutes. Their only concern or business is hunting those whites for money. When there is no money, those harlots cannot give in for a sex deal. (Male participant ID 2843)*

Some narratives also illustrated competition for resources within the peacekeeping economy. For example, the following narrative, perceived to have been initiated by the woman/girl, illustrated how women were competing with each other for MONUSCO personnel and their financial support. The peacekeepers in this narrative were perceived to be able to offer protection:

*When ladies came back, they were dressed in shorts from the pubs or brothels. The young ladies asked the agents of MONUSCO to touch their breasts and give them money in return. When a young lady accepted her breast to be touched, the prostitute forced her to move away complaining that she was receiving too few men because of her. (Female participant ID 2237)*

A few stories emerged about how sex work increased once MONUSCO arrived and how sex workers suffered financially after the peacekeepers left. This phenomenon also illustrated how compensation was linked to protection. For example, the MONUSCO personnel in following narrative were perceived by the participant as being able to offer protection:

*… MONUSCO agents have left. The majority of prostitutes have left the place, but a small number of adolescents remains among them. After a given period MONUSCO agents came back, and some prostitutes are returning as well… As MONUSCO agents have gone, prostitutes are now suffering,... (Male participant ID 542)*

#### Intermediary who facilitated the interaction

The presence of an intermediary, usually an adolescent boy, solicited by MONUSCO personnel to help facilitate sexual interactions with women/girls emerged as a regular theme, adding another nuance to the perception that peacekeepers were able to offer financial support and protection. A few narratives about transactional sex were told from the perspective of an intermediary, such as the following male participant who interpreted the peacekeeper as being able to offer protection and financial support:

*…a MONUSCO agent told me to find him a girl with whom he could make love. The first day, I called my sister, so that man gave me biscuits. The second and the third time, I called my sister. The other times, she was going there by herself, and she was coming back with money and food… She was in fact making love with him. (Male participant ID 2294)*

Intermediaries who sought out sex workers for MONUSCO personnel were compensated for their efforts. One adolescent male intermediary stated “*Sometimes these whites ask us to go and find women for them. They promise to give us 5 dollars and 20 dollars for the woman in case we succeed to bring one”* (ID 1570). The peacekeeper in the following narrative was interpreted by the participant as being able to offer protection:

*Young men or boys are used by the agents of MONUSCO to seek or show prostitutes… Do those agents of MONUSCO give them money? Oh, yes, they do give them money to buy items for them and bring them to the camp. (Male participant ID 2383)*

3. *Support-seeking*

Various support-seeking actions were described for women/girls who had interacted with UN personnel, including: (1) Mothers requesting support for their peacekeeper-fathered children and (2) Informing MONUSCO officers about the SEA. Many interactions with peacekeepers, including some sexual interactions, were perceived as offering protection as explored above. However, the potential harms and dangers were also recognized by some participants, particularly when a pregnancy resulted from a peacekeeper interaction and the mother was left with the sole responsibility of caring for the peacekeeper-fathered child or children.

#### Mothers requesting support for their peacekeeper-fathered children

Children fathered by a UN peacekeeper and born to a local mother are oftentimes abandoned once the father is redeployed, repatriated, or once his mission ends. In most cases, this leaves mothers and children living in exacerbated poverty [48, 60, 61]. In narratives involving peacekeeper-fathered children, a majority of the mothers faced abandonment from the peacekeeper father, as illustrated by broad sentiments such as “*MONUSCO white men are here for destroying by the fact that they are impregnating our young ladies and abandoning them*” (Male participant ID 483). However, several participants shared stories about support, monetary or otherwise, received for their children because of support-seeking actions. Other narratives focused on seeking support in the form of advice, most likely concerning financial assistance, from MONUSCO personnel, which added another dimension to MONUSCO personnel offering protection. The peacekeeper in the following narrative was perceived as able to offer protection:

*When the men from MONUSCO went away … the girl didn’t know who could take care of that pregnancy from the man of MONUSCO. When they arrived, she went down to see them at their base. He gave her advice about how to take care of that child.. (Female participant ID 70)*

A few participantsalso mentioned mothers advocating for food for their peacekeeper-fathered child, such as in the following narrative in which the peacekeeper was perceived as able to offer protection:

*…when she got a MONUSCO’s agent pregnancy he left her while she had already a baby … after having taken the baby’s food she comes back … after one month she goes again to take another food till [the baby] grows up and this is the way she’s living… (Female participant ID 469)*

#### Informing MONUSCO officers about the SEA

Mothers of peacekeeper-fathered children also informed MONUSCO officers about the sexual interactions that led to their pregnancies. These narratives shed light on what types of interactions were interpreted as being initiated by the woman/girl in the story. For example, the following narrative, interpreted as having been initiated by the woman/girl, described a sex worker who advocated for money from MONUSCO:

*The prostitute became pregnant and gave birth. The mother of that baby often sent us to see that man, and to ask him why he couldn’t pay a visit to his son. … Then we went to see the agent of MONUSCO very often and got the money for the baby. (Female participant ID 2273)*

In addition to providing support, informing MONUSCO about the sexual interactions occasionally resulted in the punishment of peacekeepers. For example, the following participant who interpreted the peacekeeper as able to offer financial support, described how the peacekeeper was “chased” away:

*My sister was working at MONUSCO, she fell in love with a MONUSCO black worker and he got her pregnant. Once she got pregnant, she informed her parents and both of them with the lady went straight to MONUSCO and informed MONUSCO officers about the issue. When MONUSCO authorities received a report as such, the guy was chased the same day. (Female participant ID 471)*

However, reporting SEA could also be futile for the affected women/girls. For example, the following narrative, interpreted as being initiated by the woman/girl, illustrated how in situations of rape, not being able to identify the perpetrator was detrimental for gaining support. It should be noted that although the woman/girl in the story was raped, the narrator may have perceived that the woman/girl initiated the interaction due to a variety of possible reasons, such as where the woman/girl was at the time the assault occurred or the time of day she was out.

*…she was caught by some MONUSCO guys, raped, and later delivered a white baby girl… people suspect the Pakistanis. Whenever new troops of Pakistanis come here, she always tries to go and see them in order to find her rapist. (Female participant ID 1881)*

## Discussion

This study examines adolescents’ perceptions regarding peacekeeper-perpetrated SEA in the DRC. Quantitative SenseMaker data showed that in comparison to all other age groups, male and female adolescents were significantly more likely to indicate that interactions with peacekeepers were initiated by the woman or girl, while male adolescents were more likely to perceive MONUSCO personnel as able to offer protection and to indicate that their interactions with local women/girls were sexual in nature. Three prominent qualitative themes were identified. Poverty, including a need/desire for food, education, material goods, and support for family, was central to bringing the peacekeeper and woman/girl together. Second, material/financial gain through transactional sex and sex work with peacekeepers was often used to address poverty and its related challenges. Third, support-seeking by SEA-affected women/girls was particularly common when there was a peacekeeper-fathered child, with informal reporting mechanisms sometimes leading to small amounts of food and/or money to make ends meet. As a result of the financial/material resources provided by peacekeeping personnel, both through transactional sex and sex work as well support-seeking following experiences of SEA, male adolescents were more likely to perceive peacekeepers as offering protection, contradicting the global view of SEA as harmful and in some instances criminal (those involving underage minors and rape).

Our finding that poverty was a critical underlying factor contributing to SEA in peacekeeping economies is consistent with other evidence, which has emphasized the significance of socioeconomic factors in the initiation of sexual interactions between local women and peacekeepers [48, 49, 62]. Peacekeeping economies occur due to the economic, social, and cultural impacts that peacekeeping missions have on their host communities [62, 63]. The divergent economic realities between local communities and UN peacekeepers are key, and the inconsistent nature of income-generating activities often result in local community members becoming heavily involved in the informal sector of the peacekeeping economy [63]. For instance, in the DRC, UN military personnel earn 500–1000 times the average Congolese citizen’s income, meaning they have extensive economic influence on the local population [38]. In our data the prevalence of transactional sex and sex work reflect the high involvement of women/girls in the informal peacekeeping economy and highlight the unique ways in which host community women experience feminized poverty.

Our results illustrate the relationship between sex work and the peacekeeping economy, with the competition for resources at the forefront of narratives, and sex workers suffering after peacekeepers left. Increased demand for sex work with the arrival of peacekeepers was one of the first indicators of SEA within UN PKOs in the late 1990s [31, 39, 40]. That this phenomenon was also present in our data collected in 2018 indicates the failures of UN interventions to curb SEA as a consequence of peacekeeping economies. Furthermore, the competition for resources as perceived by the adolescents in our analysis illustrates the underlying pervasive and structural poverty that motivates these types of interactions, a factor that is not easily dealt with solely by preventative training for peacekeepers.

An intermediary helping to facilitate sexual interactions between UN peacekeepers and local women/girls has not been explicitly described in the literature about aid or peacekeeping except for Csáky’s 2008 Save the Children report that mentioned peacekeepers had paid young boys to solicit girls for sex [46]. A similar concept of a “broker” has been mentioned in literature about sex work and trafficking, although brokers are typically older men or women who facilitate clients for sex workers [64, 65]. The opportunistic nature of the younger boys used as intermediaries, and compensation that the boys received, illustrates the gendered nature of peacekeeping interactions and male-dominated social norms within the DRC.

The nature of transactional sex and becoming involved in sex work—two different sexual activities albeit with sometimes unclear boundaries [59]—involves some element of agency for women. Within peace economies, local women have been perceived as “choosing” transactional activities such as sex work or transactional sex [63], and initiating a sexual encounter is a sexually agentic behaviour [66]. However, although there can be a level of agency in transactional sex that resists exploitation, and these relationships are prevalent in sub-Saharan Africa [67], others have claimed that due to inherent power differentials, any sexual interaction with peacekeepers is exploitative, rendering consent immaterial [41, 68]. Furthermore, it is extremely important to recognize that adolescents cannot consent to sexual relations and any type of sexual interaction with peacekeepers constitutes abuse, regardless of adolescents (both male and female) being more likely than other age groups to indicate that the woman/girl had initiated sexual interactions. Adolescents as an age group are more vulnerable than adults [69], which in peacekeeping and conflict settings may diminish awareness of power dynamics at play in relationships, whether of a sexual or intermediary nature. Poverty and the necessity of adolescents to meet their basic needs likely creates a complex relationship between financial resources, a sense of safety/protection, and agency in sexual interactions. Further research is needed to better understand these nuances.

Our analysis highlights that adolescents were particularly vulnerable to transactional sex and sex work in DRC’s peacekeeping economies since imbalances in power are exacerbated by the age differences between peacekeepers and host community adolescents [24, 41, 49, 67]. Additionally, adolescent girls often lack the negotiating power to protect themselves through safer sex, and a lack of sexual and reproductive health education as well as limited access to contraception, can put girls at higher risk of sexually transmitted infections, unplanned pregnancies, and intimate partner violence [67].

The male adolescent perception that peacekeepers were able to offer protection through sexual interactions must be interpreted within the context of poverty. Studies have concluded that transactional sex within the African context is often *not* perceived as exploitative [67, 70, 71]. Our results take this one step further by illustrating how transactional sex with peacekeepers was perceived as protective by males aged 13–17. Protection in peacekeeping settings is inherently gendered, in that peacekeepers situate themselves as outside and above the local who is feminized, dysfunctional, and disordered, exacerbating power differentials and creating the dichotomy of “protected” versus the male peacekeeper “protector” [63, 72]. Within this context, protection is made to be earned rather than freely given [72] and peacekeepers have reframed the exchange of sex for goods and services as a “positive, nearly altruistic intervention” [28]. These findings must also be interpreted within the context of DRC’s patriarchal society where men are expected to be the providers for their families [73].

Research has suggested that structures in peacekeeping contexts, including perceived racial hierarchies and economic disparities as well as perceptions of peacekeepers as hypermasculine 'ideal' warriors [74], result in peacekeepers seemingly outperforming local men/boys. From that perspective, it is interesting that it was male youth specifically who perceived that peacekeepers were offering protection. Additional research is needed to further examine if and how competition with peacekeepers for girlfriends and wives may have played a role in our findings. Furthermore, there were several instances in the current analysis where participants referenced race and nationality in their narratives. Other scholars have analysed race and nationality in peacekeeping contexts [74, 75]. However, since it was not the focus of our analysis, the degree to which race and nationality impacted host community members’ understanding of protection is not explored and we identify this as another important area for future research.

Our findings around support-seeking and reporting of SEA are also noteworthy. Perceptions of reporting and its efficacy varied, and barriers to reporting included not being able to identify the implicated peacekeeper, which was particularly relevant with more coercive sex or in isolated sexual encounters when the perpetrator was more likely to be unknown to the woman/girl (e.g., rape). Repercussions for peacekeepers who perpetrated SEA were rarely mentioned in the narratives, indicating that either punitive mechanisms were not effective, that peacekeeper immunity prevented punishment [35], or that repercussions were invisible to the community. Accessible protection and justice-seeking mechanisms have been an issue in peacekeeping operations globally for decades, including multiple different missions in Timor-Leste [73]. Smith [76] points out that the lack of knowledge about what happens to those accused of SEA after the initial accusation, or immediate repatriation of the accused, removes access to knowledge about repercussions and justice [73]. The issue of underreporting SEA is prevalent in all PKOs with SEA allegations [46]. However, underreporting may be particularly prevalent in the DRC, stemming from the high presence of transactional sex and cultural norms that discourage reporting abuse [33]. The likelihood of reporting SEA in cases of transactional sex and sex work may be particularly low due to the reliance on peacekeeper “clients” as a source of income for affected women and girls [33]. Furthermore, depending on the ability and willingness of children and adolescents to report abuse perpetrated against them is fundamentally flawed and does not account for their vulnerability [46]. Thus the inadequacies in existing reporting and formal support-seeking mechanisms must be addressed by the UN and other actors aiming to prevent peacekeeper-perpetrated SEA.

There are several key strengths and limitations to this analysis. Our findings do not represent the perceptions of all Congolese or Congolese adolescents. Similarly, they do not reflect the full extent of peacekeeper-perpetrated SEA or the effects of these abuses on local women/girls. Additionally, SenseMaker provides briefer narratives than traditional qualitative research and some nuances may have been missed as a result. Consequently, adolescents may have perceived interactions with peacekeepers as offering protection for some reason other than financial or material gain but we were not able to detect this in the SenseMaker data analyzed here. All data was collected in Swahili or Lingala and narratives were translated to English, leaving the possibility that nuances were lost in translation. While the nature of SenseMaker data mitigates potential researcher bias, we also acknowledge that some members of the team are from the Global North and thus the results are interpreted with our own positionality and biases. However, SOFEPADI’s input and expertise helps to mitigate these biases. This work also has some noteworthy strengths. To the best of our knowledge this is the first study to examine adolescent-specific perspectives of peacekeeper-perpetrated SEA in the DRC. This research helps highlight the voices of Congolese adolescents and informs future opportunities to research how protection and agency are interpreted by youth in peacekeeping settings. From a qualitative perspective, the sample size of 159 is large and SenseMaker allowed participants to share narratives of their own choosing, permitting their perspectives to flow naturally from lived experiences. Additionally, SenseMaker also empowered individuals to interpret their own experiences using pre-defined questions, which reduced the interpretation bias inherent in more traditional qualitative research and revealed insights that would likely have been otherwise missed. Using SenseMaker, the research was able to identify ways in which our understanding of important concepts differed from those of participants (for example, perceptions around what it means to offer protection).

## Conclusion

Our findings highlight that male adolescents experiencing poverty may perceive SEA as protective through the monetary and material support gained. Currently, SEA is addressed at the level of individual perpetrators via reporting mechanisms that require the peacekeepers’ names and are based on the belief that the endemic issue can be addressed through peacekeeper training alone [17]. However, our results indicate that such an individual approach will continue to be ineffective in the face of ongoing poverty in peacekeeping settings such as the DRC. Addressing SEA needs to be a collective effort at both the community and institutional level, including community sensitization efforts to increase awareness, particularly among adolescents, that sexual interactions with peacekeepers are exploitative and abusive. While it is important to continue developing peacekeeper training and survivor-centered reporting mechanisms, these will only be successful if implemented in conjunction with initiatives for the socioeconomic empowerment of women and girls, so that sex work and transactional sex are not perceived as necessarily “protective” mechanisms to meet basic needs. A collective effort to address SEA requires ethical, locally-driven and supported research that aims to highlight and address key issues from the perspectives of Congolese community members and demands accountability from institutional bodies. Further targeted and in-depth qualitative research is urgently needed to better understand adolescent perceptions of peacekeepers in the DRC, including how and why sexual interactions with peacekeepers might be perceived as offering protection. Our finding of male adolescents driving the conceptualization of protection in local women/girls and peacekeeper sexual interactions, as well as the use of adolescent males as intermediaries, warrants additional study to understand how gender norms influence adolescent perceptions of SEA.

## Data Availability

Original data is available upon request by contacting the corresponding author.

## Appendix 1

Narrative prompts

**Table:**

Think of a woman or girl who lives near this UN base. Share a specific story that illustrates the best or worst thing for her because of living near the base
Think of a woman or girl who has interacted with UN personnel in your community. Share a specific example of a positive or negative experience that she has had as a result of her interaction with a UN personnel
Think of a woman or girl in this community. Tell a story about how the presence of UN workers has helped or harmed her

Survey questions and possible responses

**Table:**

Question	Possible responses
*Dyads*	
*The interaction and relations you shared in the story were..	1. Entirely initiated by the foreign UN or MONUSCO personnel; 2. Entirely initiated by the woman or girl or some combination in-between
*In the story shared, the peacekeeping mission..	1. Provided the girl/woman with too much protection & safety; 2. Put the woman/girl at risk & in danger or some combination in-between
*In relation to the woman or girl in your story, those in power..	1. Did absolutely nothing to support her; 2. Provided her with too much support or some combination in-between
As a result of the interaction with the UN, the social status for the woman or girl in your story was..	1. Improved too much; 2. Diminished too much or some combination in-between
*Triads*	
*This story is about..	1. Lack of protection/governance; 2. Poverty; 3. Gender inequality or some combination in-between
*In this story, the foreign UN or MONUSCO personnel was..	1. Able to offer protection; 2. In a position of authority; 3. Able to offer financial support or some combination in-between
*What was the nature of the interaction in the story?	1. Business/transactional; 2. Voluntary; 3. Sexual or some combination in-between
In the story, it would have helped the woman or girl most to have had support from..	1. NGOs or civil society organizations; 2. The UN or MONUSCO; 3. Local chiefs & communities or some combination in-between
In this story, who was responsible for the events?	1. Individual girl/woman; 2. UN or MONUSCO; 3. Community or family or some combination in-between
The events in this story were in the best interest of..	1. Girl/woman; 2. Family; 3. UN personnel or some combination in-between

All questions were optional. Questions with statistically different responses when disaggregated by age subgroups are indicated with an asterisk.

## Abbreviations

DRC: Democratic Republic of Congo
SGBV: Sexual and gender-based violence
UN: United Nations
PKO: Peacekeeping operation
MONUC: Mission de l’Organisation des Nations Unies en République Démocratique du Congo/UN Organization Mission in the DRC
MONUSCO: Mission de l’Organisation des Nations Unies pour la Stabilisation en République Démocratique du Congo/United Nations Organization Stabilization Mission in the DR Congo
SEA: Sexual exploitation and abuse
SOFEPADI: Solidarité Féminine Pour la Paix et le Développement Intégral
