# Correction to: “It was with my consent since he was providing me with money”: a mixed methods study of adolescent perspectives on peacekeeper-perpetrated sexual exploitation and abuse in the Democratic Republic of Congo

**DOI:** 10.1186/s13031-021-00420-2

**Published:** 2021-11-15

**Authors:** Georgia Fraulin, Sabine Lee, Sandrine Lusamba, Susan A. Bartels

**Affiliations:** 1 Faculty of Health Sciences/Biomedical and Molecular Sciences, Queen’s University, Kingston, Canada; 2 Department of History, University of Birmingham, Birmingham, UK; 3 Solidarité Féminine Pour La Paix et le Développement Intégral, Beni, Democratic Republic of Congo; 4 Departments of Emergency Medicine and Public Health Sciences, Queen’s University, Kingston, Canada

## Correction to: Confl Health (2021) 15:80 https://doi.org/10.1186/s13031-021-00414-0

The authors of the original article [1] found out after publication that incorrect versions of Figure 3 and 4 were used during the publication process. The incorrect and correct figures are shown in this correction article (Figs. 1, 2, 3, 4).

**Fig. 1:**
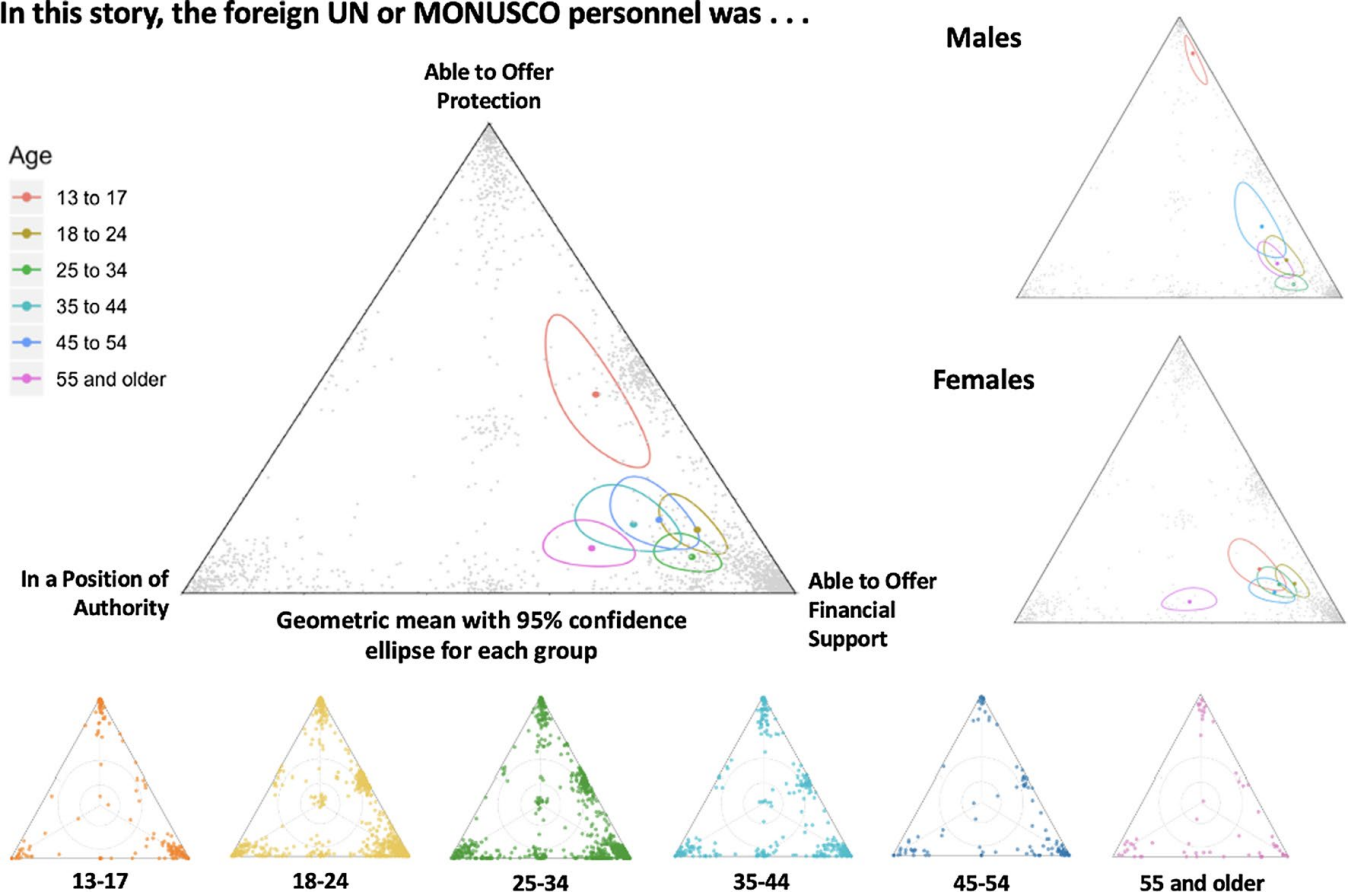
Correct version of Fig. 3

**Fig. 2:**
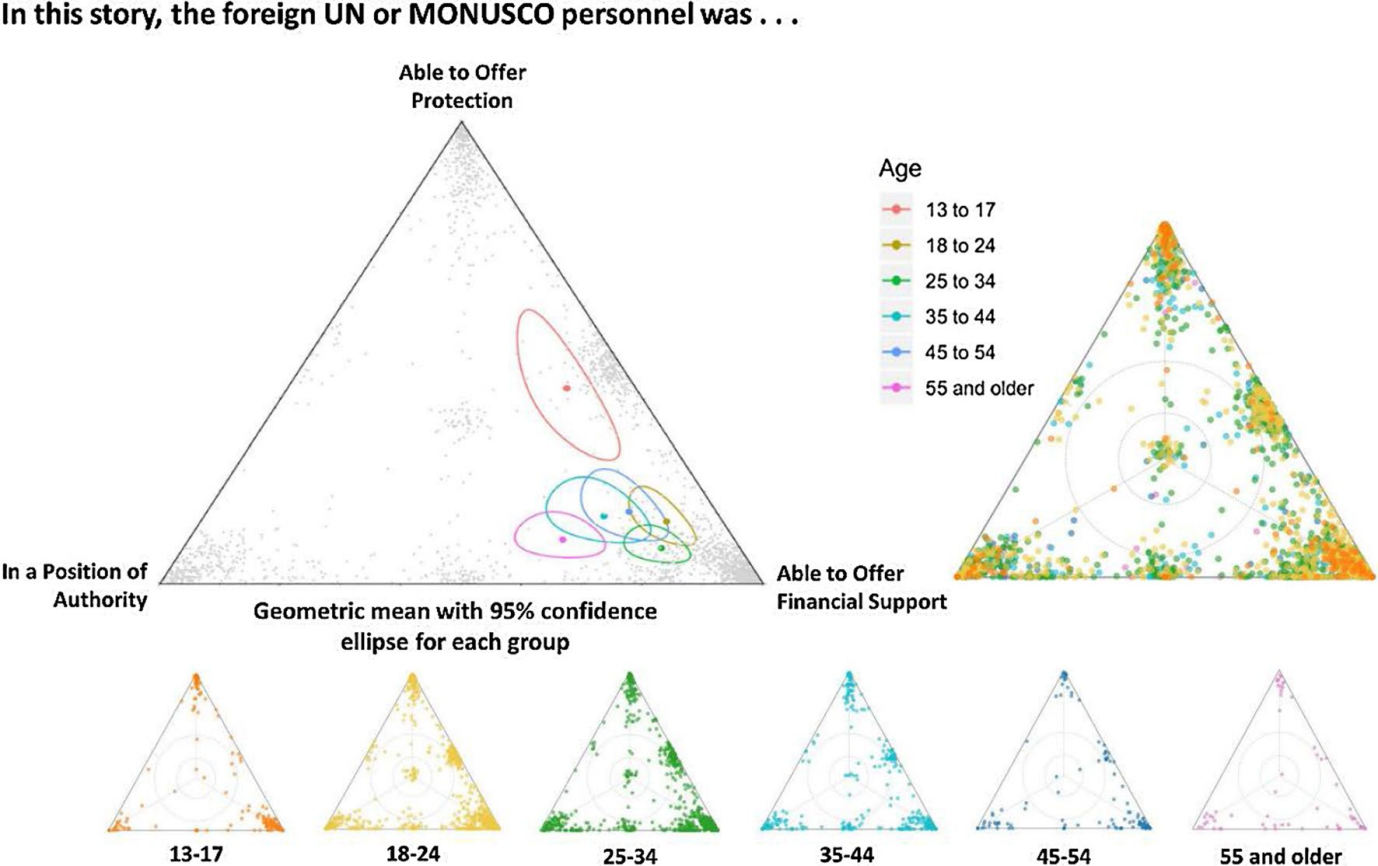
Incorrect version of Fig. 3 as originally published

**Fig. 3:**
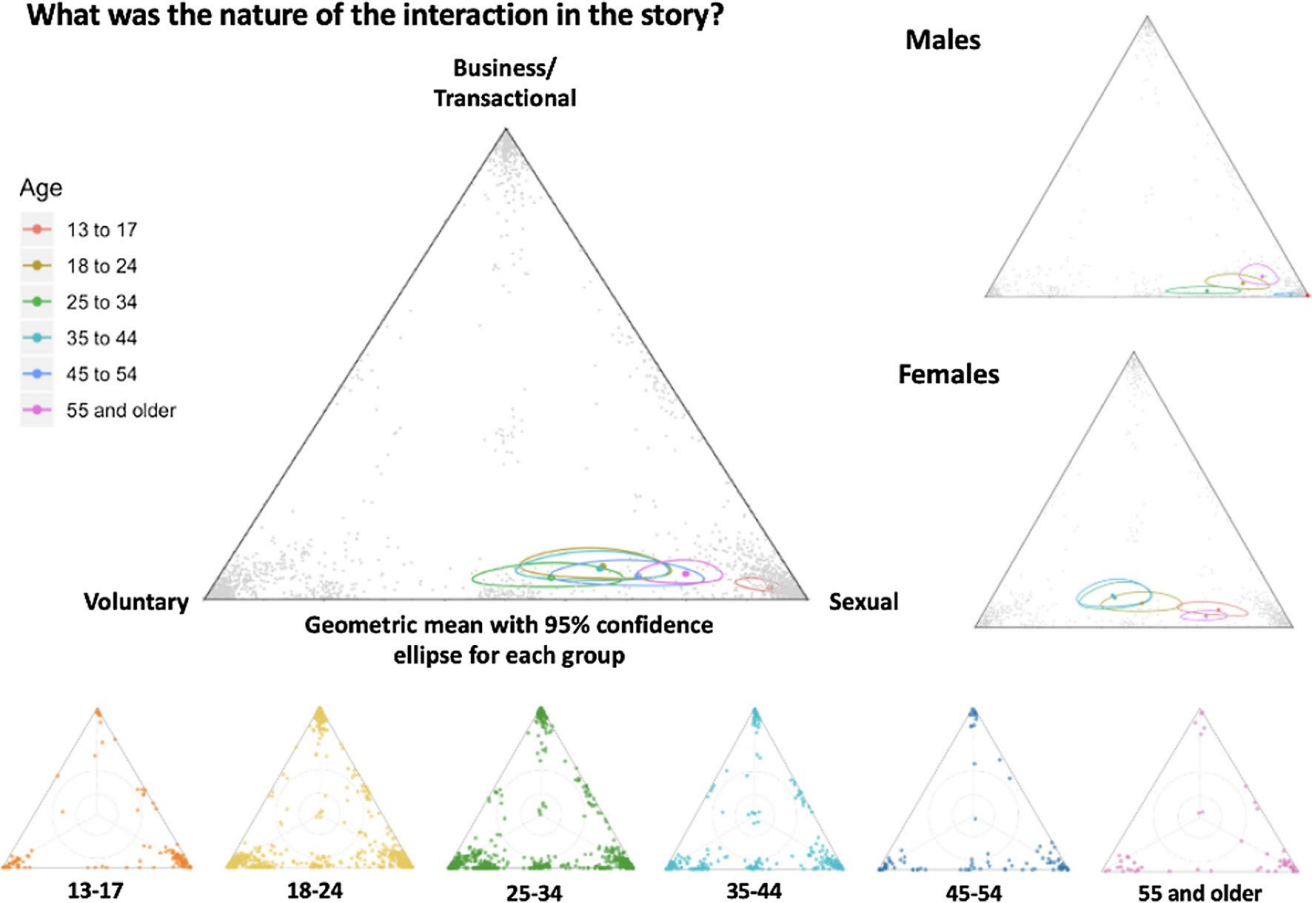
Correct version of Fig. 4

**Fig. 4:**
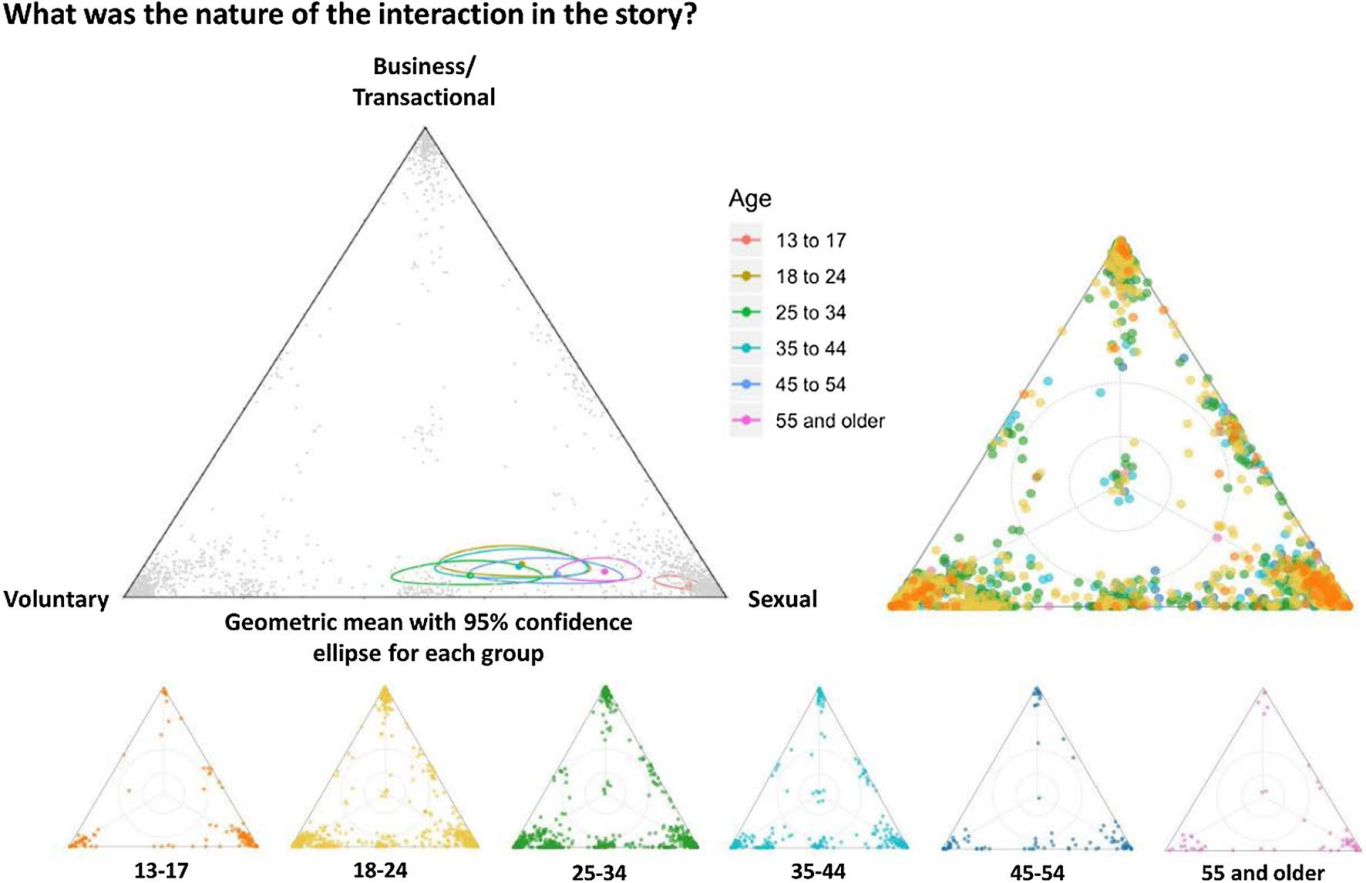
Incorrect version of Fig. 4 as originally published

This correction does not affect either results or conclusions. The original article has been updated.
